# Student satisfaction of a dementia education intervention: a cross-sectional study of the time for dementia programme

**DOI:** 10.1186/s12909-025-07218-3

**Published:** 2025-06-04

**Authors:** Stephanie Daley, Molly Hebditch, Georgia Towson, Yvonne Feeney, Sube Banerjee

**Affiliations:** 1 Centre for Dementia Studies, Brighton & Sussex Medical School, University of Sussex, Falmer, BN1 9RY UK; 2 Faculty of Medicine and Health Sciences, University of Nottingham, Nottingham, NG7 2UH UK

**Keywords:** Dementia education, Experts by experience, Healthcare students, Student satisfaction, Students, health occupations

## Abstract

**Background:**

Over the last decade there has been a recognition of the need for better dementia education for undergraduate healthcare professionals. Time for Dementia is an innovative educational programme in the UK whereby students learn about the condition directly from a two-year longitudinal contact with a person living with dementia and their families. There is evidence that such programmes have positive outcomes for students in terms of improved attitudes and knowledge, however, students’ evaluations of these programmes are scarce.

**Objectives:**

To understand the satisfaction of the students taking part in Time for Dementia and their perceptions of the programme.

**Methods:**

A cross-sectional survey, with Likert responses, was completed to assess overall levels of satisfaction for students enrolled in Time for Dementia. 1,225 students consented and completed the satisfaction survey at five universities in England. Factors that might predict satisfaction were explored using multiple regression analysis. A qualitative framework thematic analysis explored the best aspects of the programme and possible improvements, as recorded by student responses to open text questions.

**Results:**

78% of students agreed or strongly agreed that Time for Dementia had increased their knowledge of psychosocial issues, and 69% enjoyed the programme. The multiple regression analysis found satisfaction was statistically significantly higher for students who completed more visits; took part after the onset of the COVID-19 pandemic; were of Black or Asian ethnicities (compared to White British/European); and were relatively older. The themes related to the best aspects of the programme were that Time for Dementia provides relational learning, understanding the impact of dementia on family and thinking psychosocially. Improvements include preferred programme structure, the need for clarity of expectations and addressing barriers to learning.

**Conclusions:**

This study supports the value of Time for Dementia as assessed by students. Key considerations to ensure satisfaction include the fidelity of programme experience and clear expectations.

**Supplementary Information:**

The online version contains supplementary material available at 10.1186/s12909-025-07218-3.

## Background

Improving dementia care is a global imperative. An increasing number of countries have responded in the form of national dementia strategies [1–5] but there continues to be evidence of suboptimal care and the need for action [6, 7]. A key component to improving dementia care is improving education and training of the healthcare workforce in dementia [6–9].

Understanding how to best train healthcare professionals is therefore a priority. Research suggests that crucial elements include: ensuring that dementia education is introduced early in the career pathway; encouraging reflection on the lived experiences of dementia such as simulation or direct contact with people with dementia; and avoidance of over-reliance on traditional didactic lectures [10, 11]. At the undergraduate level, the objectives of training should focus on facilitating positive attitudes and holistic knowledge of dementia, as well as increasing confidence and willingness to work with people with dementia once qualified [8, 12–14]. These objectives are vital to instil in students early into their training when students’ attitudes and opinions are more malleable.

Time for Dementia is an example of an innovative dementia educational programme aiming to address some of the limitations of current dementia undergraduate education. In Time for Dementia pairs of students meet a family (a person with dementia and their carer) for 2 h at the family’s home, 6 times over 2 years, supported by other learning activities. Students use visit guides to facilitate informal but focused visits around learning about the family experience. Students are guided by a Time for Dementia handbook and dedicated administrators. The families who volunteer for the programme are supported by Alzheimer’s Society. A protocol paper by Banerjee et al., (2017) describes the rationale and implementation of the programme in more detail [15]. Time for Dementia is unique in its scope for being designed to be delivered to all healthcare professional groups in training and for being a mandatory component of the curriculum. Since its establishment in 2014, it has been delivered to over 8,000 medical, nursing, paramedic and allied health profession students and is currently running at nine universities in England.

Prior research on innovative dementia education using objective standardised measures of learning outcomes has provided evidence they can positively influence attitudes and knowledge towards dementia [12, 13], including Time for Dementia [16]. However, there are limited data on students’ subjective evaluations of these programmes. Analyses of student journal reflections and qualitative interviews have been used to assess perceptions of learning, which have been positive [17–20]. However, there is little research on student opinions of programme components [21, 22] or more generalisable quantitative data analyses on satisfaction.

A previous mixed methods evaluation of Time for Dementia has investigated student outcomes (attitudes and knowledge) [16, 23], student learning mechanisms [20, 24], family experiences [25], implementation factors [26], and long-term outcomes (ongoing); this current study explores student satisfaction. Evaluating student satisfaction is the foundational pillar in research on educational interventions [27]. Understanding levels of satisfaction is important because: (i) higher satisfaction can facilitate learning, (ii) students’ perceptions of their learning can add validity to evidence of learning, and (iii) it allows the inclusion of the student’s voice in the development of educational interventions. This study aims to understand the satisfaction of the students taking part in Time for Dementia and their perceptions of the programme.

### Research questions

1. How satisfied are students with their experience of Time for Dementia?
2. What factors are associated with satisfaction in Time for Dementia?
3. What are students’ perceptions of their experience in the Time for Dementia programme?

## Methods

### Design

This is an analysis of quantitative and qualitative data collected from 2014 to 2021 as part of the Time for Dementia evaluation [15] which is in the form of a mixed methods longitudinal cohort study. All universities taking part in Time for Dementia during this time period were included in the evaluation. Data for the wider evaluation were collected from participants before their first Time for Dementia visit (baseline) and approximately 24 months later (follow-up) after the students had completed the programme. This paper presents the results of the cross-sectional satisfaction survey (administered at follow-up only), as well as demographic information (collected at baseline). Dementia specific patient and public involvement was sought at all key stages, including the initial design of the study and choice of measures, and at points of key amendments. This was facilitated through a dementia research advisory group, family members, people with dementia, and the Alzheimer’s Society also actively participated in the Time for Dementia Executive Board.

### Study setting and sample

Participants included healthcare students enrolled in Time for Dementia as part of their mandatory curriculum. The programme was delivered to nursing, medical, paramedic and allied health professional students (including occupational therapy, physiotherapy, speech and language therapy and radiology). This study examined 24 cohorts of students at five universities in the South of England.

Students visited their paired family 5–6 times over two years. All students were monitored to complete visits and required to attend a preparatory workshop and final reflective session. Some differences in implementation occurred due to curriculum fit; the programme started in either the first or second year of training, and may have had a linked assignment. Furthermore, students’ number of completed visits was influenced by disruption to the programme through either family withdrawal from the programme or COVID-19 disruption. All students who were required to participate in the Time for Dementia programme were eligible to take part in research regardless of visits completed.

### Procedure

Participation in the evaluation of Time for Dementia was voluntary, students were invited to take part during scheduled lectures. They were given an information sheet in advance, and written informed consent was obtained. Students’ surveys were anonymised with a participant identifier by the research team that allowed surveys to be matched.

In March 2020, the COVID-19 pandemic led to the cessation of face-to-face visits between students and Time for Dementia families. The student cohorts affected by this disruption had a reduced number of visits and their last contact with their allocated families was conducted over the telephone. For the analysis, this disruption was recorded into two groups: those who completed the programme and their 24-month follow-ups before 1st March 2020 (no disruption) and those who completed after this date (possible disruption).

### Measures

Students completed a questionnaire including demographic information at baseline and a satisfaction survey at follow-up, after completing the programme. Measures were completed as hard copy questionnaire packs, or online using Qualtrics (Provo, UT) where in-person meetings were not possible.

The satisfaction survey was adapted from the version used to evaluate *The Buddy Program* [28], a dementia educational initiative that informed the development of Time for Dementia [15]. The modified survey was a 12-item questionnaire (available in Supplementary File 1). It was divided into two sections: one assessing student *satisfaction with the organisation* of the programme (Q1,2,3,4,10) and the other evaluating *satisfaction with learning outcomes* (Q5,6,7,8,9,11,12). Satisfaction was rated using Likert scales ranging from one = strongly disagree, to five = strongly agree, with higher scores indicating greater satisfaction. One question (Q3) could be interpreted either as a positive or negative evaluation of the programme; after reviewing accompanying qualitative responses and correlations with other items it was decided to be coded as higher agreement as higher preference, in line with other items for analysis. Two open text questions were included:

- “What were the BEST aspects of the Time for Dementia programme?”
- “What IMPROVEMENTS could be made to the Time for Dementia programme?”

### Analysis

Summary statistics are reported for demographic information and frequencies of responses for satisfaction items. Thematic framework analysis [29] was completed for the participant responses to open text questions. The framework was developed following a previous analysis of interviews with students taking part in Time for Dementia [20]. The transcripts were coded using Excel (v1808) by GT. Each meaningful unit of text was coded with a descriptive code. Codes were discussed in detail under the supervision of an experienced qualitative researcher (SD) to enhance validity and reflexivity.

Total satisfaction scores for each survey section (organisation, and learning outcomes) were calculated. Satisfaction surveys were excluded if more than 20% of items were missing in each subsection, otherwise person-mean imputation was used. Total scores had acceptable reliability for the two sections (Cronbach’s α = 0.66 and 0.89). In a multiple regression model factors that might predict satisfaction in students for both subsections of the satisfaction survey were analysed. Possible predictors included sex (male vs. female), age, ethnicity, student type, number of visits, prior experience of dementia (yes vs. no) and COVID-19 disruption (yes vs. no). Statistical significance was defined as a *p*-value < 0.05. Assumptions for linear regression were examined, and robust standard errors were calculated as residuals deviated from normality. All data were analysed using Stata 17.0 (Stata Statistical Software: Release 17, StataCorp LLC) and figures were produced in SPSS (V.26).

## Results

### Response rate

3,619 students were eligible to take part in the study. 2,700 (74%) consented to take part and completed baseline demographics. From the consenting group, 1,225 (45%) contributed to follow-up data on completing the programme. This paper presents data from the follow-up sample only.

### Demographics

Student demographics are presented in Table 1.

**Table 1:** Participant demographics and characteristics at baseline

	*n*	%
**Age (years)**	21.0 (median)	19.0 to 28.0 (IQR)
**Type of student**		
Medical	394	32.2
Nurse (adult or mental health)	415	33.9
Paramedic	135	11.0
Allied Health Profession	281	22.9
Total	**1**,**225**	**100.0**
**Student Sex**		
Female	916	75.6
Male	295	24.4
Total	**1**,**211**	**100.0**
**Student Marital Status**		
Never Married	866	75.5
Currently Married	156	13.6
Cohabiting	78	6.8
Separated/Divorced	46	4.0
Widowed	1	0.1
Total	**1**,**147**	**100.0**
**Student Ethnicity**		
White British/European	884	76.3
Mixed/Multiple Ethnic Groups	34	2.9
Asian/Asian British	114	9.8
Black/African/Caribbean/Black British	99	8.5
Other	28	2.4
Total	**1**,**159**	**100.0**
**Experience of knowing someone with Dementia**		
Yes	606	52.4
No	550	47.6
Total	**1**,**156**	**100.0**

The number approached for research could not be calculated as registers for attendance at recruitment sessions and the numbers approached via email were not available. Therefore, the number presented for eligible students is based on the total intake numbers of student cohorts

### How satisfied are students with their experience of time for dementia?

**Fig. 1:**
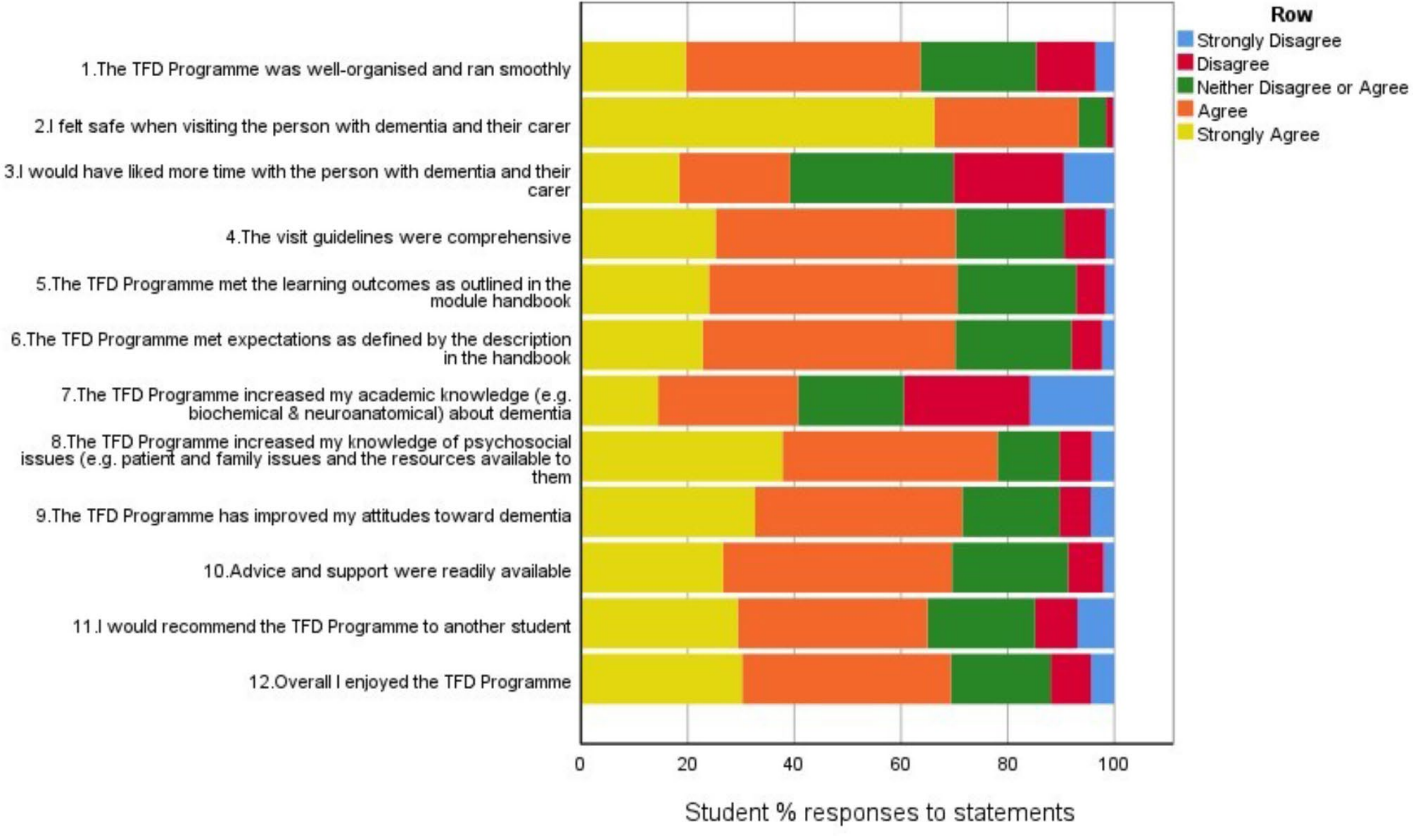
Frequency of responses (%) to items on the satisfaction survey (*n* = 1,177)

Figure 1 presents the results of the satisfaction survey as frequency of responses by students to each item. The two items students most frequently agreed or strongly agreed with (indicating areas most satisfied with) were that they felt safe during the visits (93.3%) and that the Time for Dementia programme had increased their knowledge of psychosocial issues (78.1%). The majority agreed that they enjoyed the programme (69.3%) and would recommend other students to take part (65%). Overall, at least 60% of students either strongly agreed or agreed with ten out of the twelve items, indicating that the majority of students evaluated Time for Dementia favourably. The two items with the least agreement were that they would have liked more time with the families and that the programme had increased their academic knowledge of dementia, with 39.2% and 40.8% agreeing or strongly agreeing with these statements respectively.

### What factors are associated with satisfaction in time for dementia?

Table 2 presents the student total satisfaction scores, and number of visits and COVID-19 disruption as recorded at the end of the programme.

**Table 2:** Total satisfaction scores and number of visits at follow-up

		COVID-19 disruption NO			COVID-19 disruption YES		Total	
		No.	%		No.	%	No.	%
Number of visits								
**0**		2	0.3		0	0	2	0.2
**1**		13	1.8		37	8.1	50	4.2
**2**		58	8.0		43	9.4	101	8.6
**3**		94	13.0		79	17.4	173	14.6
**4**		161	22.2		121	26.6	282	23.9
**5**		265	36.5		84	18.5	349	29.6
**6**		132	18.2		91	20.0	223	18.9
**Total**		755	100		455	100	1,180	100
	**Mean** (**SD**)			**n**	**Mean** (**SD**)	**n**	**Mean** (**SD**)	**n**
**Total satisfaction (12–60)**	44.5 (8.4)			708	46.3(8.8)	475	45.0(8.6)	1,183
**Satisfaction for Organisation** **(5–25)**	18.8(3.2)			709	19.6(3.2)	475	19.1(3.2)	1,184
**Satisfaction for Learning outcomes (7–35)**	25.7(5.7)			703	26.7(6.1)	475	26.2(5.9)	1,178

Table 3 presents the results of the multiple regression models for predictors of satisfaction with the organisation of the programme. There was strong evidence of an association with higher satisfaction and a higher number of visits (coefficient: 0.40, 95% confidence interval [95% CI]: 0.23–0.58, *p* < 0.001), older age of students (0.04, 95% CI: 0.01–0.06, *p* = 0.006) and those completing the programme after 1st March 2020 (COVID-19 pandemic). There was also evidence for a positive association with ethnicity for Black/ African/Caribbean/Black British compared to White British/European (0.87, 95% CI: 0.14–1.61, *p* = 0.020). No association was found with participant sex, prior experience of dementia, or student type.

**Table 3:** Predictors of student satisfaction for the organisation of time for dementia (*n* = 1,038)

Variables	B	Lower 95% CL	Upper 95% CL	*p*
**(Constant)**	16.27	14.96	17.59	< 0.001
**Student Sex (Male vs. Female)**	-0.26	-0.72	0.21	0.280
**Student Age**	0.04	0.01	0.06	0.006
**Student Ethnicity (compared to White British/European)**				
Mixed/Multiple Ethnic Groups	-0.46	-1.49	0.58	0.385
Asian/Asian British	0.55	-0.02	1.12	0.058
Black/African/Caribbean/Black British	0.87	0.14	1.61	0.020
Other	0.23	-1.51	1.96	0.799
**Student Type (compared to medics)**				
Nurse	-0.04	-0.60	0.51	0.879
Paramedic	-0.40	-1.16	0.37	0.310
Allied Health Profession	-0.49	-1.14	0.16	0.137
**No. of Visits completed (0–6)**	0.40	0.23	0.58	< 0.001
**Experience of Dementia (Yes vs. No)**	-0.05	-0.46	0.35	0.803
**COVID-19 disruption (Yes vs. No)**	1.12	0.68	1.56	< 0.001

Table 4 presents the results of the multiple regression models for predictors of satisfaction for learning outcomes of Time for Dementia. Again, there was strong evidence of an association with higher satisfaction and a higher number of visits (0.76, 95% CI: 0.45–1.08, *p* < 0.001), and completion after 1st March 2020 (1.48, 95% CI: 0.71–2.26, *p* < 0.001). There was also evidence for a positive association with ethnicity for Black/African/Caribbean/Black British (2.70, 95% CI: 1.51–3.89, *p* < 0.001) and Asian/Asian British (1.99, 95% CI: 1.09–2.89, *p* < 0.001) compared to White British/European. There was no evidence to support an association with participant sex, age, the experience of dementia and student type.

**Table 4:** Predictors of student satisfaction for learning outcomes of time for dementia (*n* = 1,032)

Variables	B	Lower 95% CL	Upper 95% CL	*p*
**(Constant)**	20.78	18.43	23.13	< 0.001
**Student Sex (Male vs. Female)**	-0.05	-0.93	0.84	0.919
**Student Age**	0.01	-0.03	0.06	0.556
**Student Ethnicity (compared to White British/European)**				
Mixed/ Multiple Ethnic Groups	0.31	-1.30	1.93	0.704
Asian/Asian British	1.99	1.09	2.89	< 0.001
Black/African/Caribbean/Black British	2.70	1.51	3.89	< 0.001
Other	0.99	-1.94	3.91	0.508
**Student Type (compared to medics)**				
Nurse	0.58	-0.41	1.58	0.252
Paramedic	-0.73	-2.34	0.89	0.376
Allied Health Professional	-0.08	-1.26	1.09	0.890
**No. of Visits completed (0–6)**	0.76	0.45	1.08	< 0.001
**Experience of Dementia (Yes vs. No)**	0.45	-0.27	1.17	0.223
**COVID-19 disruption (Yes vs. No)**	1.48	0.71	2.26	< 0.001

### What are students’ perceptions of their experience in the time for dementia programme?

1,032 students responded to the open text questions on the survey providing their best aspects, and 971 students suggested improvements. The best aspects of the programme were categorised into six key themes, and improvements were categorised into eight key themes, presented in Table 5.

**Table 5:** Best aspects and improvements of time for dementia

Best aspects themes (% Freq of theme)
**Relational Learning (57.97%)**
*‘Hearing life stories and bonding with a person I would otherwise never have engaged with. Learnt more about how I could help carers if I were their doctor of person with dementia.’ P035* *‘Lovely family - nice to build up a long term rapport.’ P087* *‘Long term development of a relationship with a patient + family*,* learning details of how their lives have been affected day to day’ P123*
**Understanding the impact of dementia on family (22.94%)**
*‘Chance to learn from a person with dementia and their caregiver - I have come away from each session wanting to know more and gaining insight into the family’s lived experience has been so beneficial’ P434* *‘Being able to visit a person with dementia on multiple occasions over a period of time allowed us to observe the changes that occur as disease progresses’ P953* *‘Getting a detailed first hand story of all the challenges a family living with dementia face was very beneficial. The family and carers gave great insight into the daily issues they face*,* some if which would not be thought about* (by student learner).*’ P985*
**Thinking Psychosocially (7.97%)**
*‘See how people and their families live and cope with dementia gaining an understanding of what they are going through in order to inform my clinical practice.’P462* *‘Improving my understanding of psychosocial issues for patient and family.’ P078* *‘Particularly talking with the carer allowed me to understand some of the more “non medical” symptoms and experiences of the disease*,* which gave me better insight and a more holistic understanding of dementia.’P738*
**Enhanced Dementia Practice (7.01%)** *‘Hearing points of view that will shape how I communicate going forth’ P909* *‘Being comfortable communicating with dementia patients’ P029* *‘Boosted my autonomy*,* confidence’ P220*
**Carer offload/feeling a purpose (2.59%)** *‘The carer genuinely enjoyed the visits and it was lovely to chat to her and feel like we could act as a listening ear’ P980*
**Unable to identify best part (1.34%)** *‘nothing’ P206*
**Challenging Attitudes (0.19%)** *‘It allowed to open my mind to a lot of misconceptions I had’ P456*
**Suggested improvement themes (% Freq of theme)**
**Preferred programme structure (35.48%)**
*‘Maybe spending more time with the family. Having more visits. And perhaps having another family as well to visit. That will increase the exposure to different families’ P021* *‘3 visits a year seemed to be a lot*,* especially during all other challenges university brings*,* and when it was 40 miles from campus*,* very time consuming.’ P403* *‘Better timings to work around our schedules. Set dates made with families so it is already planned out for everyone’ P424*
**Clarity of Expectations (19.39%)**
*‘More structured guidelines for what we needed to learn from the programme.’ P158* *‘Better support for the family*,* they didn’t really know what and how long we were there for.’P257*
**Barriers to Learning (19.11%)**
*‘May be useful programme for those who have not had experience in setting involving dementia patients’ P272* *‘I only got 3 visits due to the people with dementia having health problems and/or dropping out (changed 3 families altogether). So*,* I feel like I missed out on some valuable experience.’ P977*
***Travel Time (7.19%)*** *‘Sometimes the journey would be very long & tiring for us.’ P182*
**Student Discomfort (3.50%)** *‘If we have to complete this*,* it would be better as a lesson or group activity. It was very uncomfortable visiting someone in their own home.’ P214*
**Research related (1.70%)** *‘Less questionnaires.’ P355* **Beginning and End (1.23%)** *‘Clearer guidance for families/us about what to do regarding contact after the end of the programme as our family were expecting to keep in touch for a long while.’ P919*
**No Improvements (12.39%)** *‘Nothing everything was really useful!!’ P024*

The most frequent theme for best aspects was *relational learning (58%)*. Which is learning centred on ‘real life’ experiences in the context of relationships. The relationship with the family was the most valued component to students, including how ‘real life’ learning is early absorbed and retained (compared to lectures), and is facilitated by the long-term nature of the programme and real-life setting at the family’s home. Second to this in frequency (23%) was the theme of *understanding the impact of dementia on the family* which includes understanding the wider context of living with dementia, including the carers’ perspective and the global impact in relation to professionals and services. None of the remaining four themes was present in more than 10% of responses.

The most frequently cited theme for improvements was *preferred programme structure* (35%). This relates to improved consultation with students about the practical aspects of how the programme is delivered. This included recommendations for: better organisation and support in arranging visits and communication; altered visit length and frequency (more/less); and difficulty around linked assignments or pressure of the curriculum (particularly in medical students).

Joint second, was the *clarity of expectations* (19%) and *barriers to learning* (19%). Students wanted further clarity and guidance on the structure of the visits, relevance to the course and the families’ understanding of the programme. Learning was impeded by disruption to the programme (including COVID-19 disruption) and with either having prior experience (little to learn) or no prior experience (wanting more preparation and training before the visits). Students also commented they had no improvements to suggest (12%). The four remaining themes were cited in led than 10% of responses.

## Discussion

### Key findings

It is positive that 78.1% of students felt they had learned more about the psychosocial aspects of dementia care, which is one of the primary aims of the programme. This contrasts with the student perception of increased knowledge, which was lower (40.8%) and adds discriminative validity. Prior research on Time for Dementia has shown an increase in attitudes and knowledge for students who completed Time for Dementia compared to controls [16, 23] which supports the students’ evaluations. The qualitative comments also support the perceived value of Time for Dementia, with some students attributing it to considerable influences on their learning (as shown in the themes *relational learning* and *understanding the impact of dementia on family*). This is supported by previous qualitative research on Time for Dementia [20, 24] that found meaningful changes in dementia attitudes, understanding, and person-centred practice. Similar innovative educational interventions that include direct contact with people with dementia also highlight how this approach could show positive student learning outcomes, such as *The Buddy Programme* [28], *a Friend for Rachel* [30] and specialised placements [31]. Future research should evaluate if this learning is sustained and whether students still perceive an impact on their practice once qualified [13].

However, some students’ experiences were less positive. The qualitative analyses suggest areas of dissatisfaction include: burden related to time, travel and organisation issues; barriers to learning due to the perceived suitability of the programme to individuals, reduced visits or disrupted programme experience; and lack of clarity on aims or structure of the programme. This may in part be explained by implementation issues when the programme was newly introduced at each of the universities and may abate over time. For example, the suggestions for more structure or guidance for visits for students were mirrored in families’ recommendations for improvement [25]. This resulted in changes to the implementation of Time for Dementia for later cohorts included in the analysis, including further structure and guidance as well as clarity on student learning objectives. However, an analysis of changes over time was not undertaken. These findings suggest that ensuring a positive student experience of the programme is important for student satisfaction, and although some disruptions are inevitable (e.g. family circumstance change, COVID-19 etc.) administration support and clear operational procedures can reduce disruption and burden to students.

The factors associated with higher satisfaction were student ethnicity (Asian/Asian British and Black/African/Caribbean/Black British compared to White British European), taking part in Time for Dementia during COVID-19, student age, and the number of visits completed. One possible explanation for the difference in ethnicity includes a potential cultural differential interest in working with, or attitudes towards, older people and people with dementia [32] and therefore overall interest in completing Time for Dementia, although research is lacking on differences by ethnicity in the UK.

The result that satisfaction was higher in students completing during COVID-19 appears counterintuitive as the programme was disrupted during this period. Possible explanations include that students may be more likely to be lenient on their appraisal of Time for Dementia considering all education was severely disrupted. Alternatively, as discussed in the previous section, it could be confounded by time, as those completing Time for Dementia before COVID-19 would have been the first groups to take part when the programme was introduced at each university, where there may have been more administrative teething problems and established guidance. Further research is needed to understand these unanticipated findings.

Student satisfaction was higher in those who had completed a higher number of visits; this could be explained as these students are less likely to have had any disruption to their Time for Dementia experience and were able to visit the same family over time, something that the qualitative findings in this study and previous work have shown to be of core value to students [20]. This finding is also reflected in an analysis of family satisfaction with more visits associated with higher satisfaction of families [25]. While it might be suggested that satisfied students do more visits, the programme was compulsory and students were monitored to complete visits, therefore minimising such potential bias.

There was no evidence satisfaction was associated type of healthcare student despite some qualitative findings suggesting that there were some specific concerns for particular student groups. Different professional groups may have different considerations but not overall satisfaction. Variation could be explored further to optimise the program for each healthcare profession’s curriculum.

### Key strengths and limitations

The high loss to follow-up was due to students leaving the course, lack of attendance at data collection sessions, COVID-19 disruption, and perhaps apprehension at questionnaire length (as stated in student feedback). However, with 1,225 students completing the survey at five universities, this represents a large-scale study in healthcare educational literature.

Due to the low response rate there may be non-response bias in the sample, for instance, students more satisfied or more dissatisfied with the programme (or both) may have been more likely to complete the survey and there was a reduced response rate for those completing during the COVID-19 disruption. In general, the COVID-19 pandemic led to unanticipated complexity in the interpretation of these findings due to disruption to the Time for Dementia programme and wider teaching as well as research recruitment. Furthermore, there are possible confounders not accounted for in the analysis, such as changes in the programme over time, and individual variability in processes and procedures at different universities. Taken together, this limits the strength of conclusions drawn from the multiple regression analysis.

It appears these findings represent relatively high satisfaction for a student group with diverse educational preferences on a mandatory curriculum component, however understanding the magnitude of student satisfaction is difficult as there are no suitable comparison data. Yet the finding that the majority of students report positive learning outcomes is supported by research on objective measures of attitudes and knowledge [16, 23]. Indeed, the value of these results is increased by being a key part of a wider mixed methods evaluation of Time for Dementia. Lastly, this paper adds to the literature on satisfaction in experiential dementia educational programmes, a component of evaluations often missing.

## Conclusions

These results indicate that Time for Dementia is evaluated positively by students. It adds to a growing body of research that suggests innovative dementia educational programmes that draw on people with dementia as ‘experts by experience’, such as Time for Dementia, have the potential to improve the education of healthcare professionals.

## Electronic supplementary material

Below is the link to the electronic supplementary material.

Supplementary Material 1

## Data Availability

The datasets used and/or analysed during the current study are available from the corresponding author on reasonable request.

## Declarations

### Abbreviations

N/a.
