# Supplementary material for: Student satisfaction of a dementia education intervention: a cross-sectional study of the time for dementia programme
Source: BMC Med Educ. 2025 Jun 4;25:838. doi: 10.1186/s12909-025-07218-3 (PMC12139129; doi:10.1186/s12909-025-07218-3)
Supplement: Supplementary file 1 — Supplementary Material 1 [file 12909_2025_7218_MOESM1_ESM.docx]

**Student Satisfaction Survey**

We are evaluating the outcome of the Time for Dementia Programme. The questions below ask about your experience and opinions about the Time for Dementia Programme. Your responses are confidential and will not be shared with others.

1. I am a medical student/adult nursing student/mental health nursing student/paramedic student/occupational therapy student/speech and language therapy student/physiotherapy student/radiography student *(delete as appropriate)*
2. I am in the 1^st^/2^nd^/3^rd^ year of my undergraduate education *(delete as appropriate)*
3. Gender: ____________ Age: ______________
4. To date how many times have you visited the person with dementia and their carer?

_________ times

1. Did your experience in the Time for Dementia Programme challenge any misconceptions you may have had prior to participating, either about dementia or living with a long term illness?

⬜ Yes

- No

Please explain:

1. Please assess the following statements based on your experiences of the Time for Dementia Programme using the scale: 1 = strongly disagree, 2 = disagree, 3 = neither disagree or agree, 4 = agree, 5 = strongly agree.

| The Time for Dementia Programme was well-organised and ran smoothly | **1** | **2** | **3** | **4** | **5** |
| --- | --- | --- | --- | --- | --- |
| I felt safe when visiting the person with dementia and their carer | **1** | **2** | **3** | **4** | **5** |
| I would have liked more time with the person with dementia and their carer | **1** | **2** | **3** | **4** | **5** |
| The visit guidelines were comprehensive | **1** | **2** | **3** | **4** | **5** |
| The Time for Dementia Programme met the learning outcomes as outlined in the module handbook | **1** | **2** | **3** | **4** | **5** |
| The Time for Dementia Programme met expectations as defined by the description in the handbook | **1** | **2** | **3** | **4** | **5** |
| The Time for Dementia Programme increased my academic knowledge (e.g. biochemical & neuroatomical) about dementia | **1** | **2** | **3** | **4** | **5** |
| The Time for Dementia Programme increased my knowledge of psychosocial issues (e.g. patient and family issues and the resources available to them) | **1** | **2** | **3** | **4** | **5** |
| The Time for Dementia Programme has improved my attitudes toward dementia | **1** | **2** | **3** | **4** | **5** |
| Advice and support were readily available | **1** | **2** | **3** | **4** | **5** |
| I would recommend the Time for Dementia Programme to another student | **1** | **2** | **3** | **4** | **5** |
| Overall I enjoyed the Time for Dementia Programme | **1** | **2** | **3** | **4** | **5** |

1. What were the BEST aspects of the Time for Dementia Programme?

1. What IMPROVEMENTS could be made to the Time for Dementia Programme?

**Thank you for completing this form**

**To be completed by the Researcher**

Participant ID: Date:
